# Association Between Serum Vitamin D Levels and Colorectal Carcinoma: Insights from a Case Control Study in Northern Saudi Arabia

**DOI:** 10.3390/life16030512

**Published:** 2026-03-20

**Authors:** Anass M. Abbas, Ashok Kumar Sah, Reef A. Alodhayd, Shahad A. Alblehed, Aryaf M. Almaeen, Saja T. Almadhor, Hala E. Sabaa, Rania Z. Alghafil, Nasir A. Nour, Abdulkhakov Ikhtiyor Umarovich, Ranjay Kumar Choudhary, Rabab H. Elshaikh, Manar G. Shalabi

**Affiliations:** 1 Department of Clinical Laboratory Sciences, College of Applied Medical Sciences, Jouf University, Sakaka 72341, Saudi Arabia; reefabdullah3315@hotmail.com (R.A.A.); shalblaihed@gmail.com (S.A.A.); saryaf024@gmail.com (A.M.A.); sajalmadhoor@outlook.sa (S.T.A.); ehala269@gmail.com (H.E.S.); rania2782002@gmail.com (R.Z.A.); dr.mpathology@gmail.com (M.G.S.); 2 Department of Medical Laboratory Sciences, College of Applied and Health Sciences, A’ Sharqiyah University, Ibra 400, Oman; ranjay.kumar@asu.edu.om (R.K.C.); rabab.mahmoud@asu.edu.om (R.H.E.); 3 Department of Histopathology, Prince Mutaib Bin Abdulaziz Hospital, Sakaka 72341, Saudi Arabia; nournasir@hotmail.com; 4 Department of Faculty and Hospital Therapy, Bukhara State Medical Institute, Bukhara 210118, Uzbekistan; mironsho@rambler.ru

**Keywords:** colorectal cancer, Vitamin D deficiency, adenocarcinoma, clinicopathological features, case–control study, Saudi Arabia, Al Jouf

## Abstract

Background: Colorectal cancer (CRC) is a major global health concern and a leading cause of cancer-related mortality. In Saudi Arabia, it is the most common cancer among men and the third most common among women. The disease affects predominantly older adults, with an increasing number of cases reported in younger populations. Emerging evidence suggests a potential association between Vitamin D deficiency and CRC risk and progression. Aim: This study aimed to investigate the relationship between serum Vitamin D levels and colorectal cancer, and to evaluate its association with clinicopathological characteristics. Methodology: A retrospective case–control study was conducted on newly diagnosed CRC patients between January 2021 and August 2024 at King Abdul-Aziz Specialist Hospital, Prince Muteb Hospital, and the Oncology Center in Al Jouf, Saudi Arabia. A total of 100 CRC cases and 50 healthy controls were included. Serum 25-hydroxyvitamin D levels were measured and categorized as deficient (<20 ng/mL), insufficient (21–29 ng/mL), and normal (≥30 ng/mL). Histopathological features and tumor characteristics were analyzed. Statistical analyses included independent *t*-test, one-way ANOVA, and chi-square tests. Results: During the four-year period, 5399 gastrointestinal specimens were analyzed, of which 2111 (39.1%) were colorectal specimens. CRC was diagnosed in 107 cases (5.1%), and 100 patients met the inclusion criteria. The mean age of patients was 53.07 ± 13.3 years, and 69% were older than 50 years. Males represented 58% of cases (male-to-female ratio 1.4:1). Invasive adenocarcinoma was the predominant histological subtype (81%), with the sigmoid colon being the most common tumor site (39%). Vitamin D deficiency was significantly more prevalent in CRC patients (59%) compared to controls (22%). The mean serum Vitamin D level was significantly lower in cases (18.7 ± 11.3 ng/mL) than in controls (34.9 ± 15.6 ng/mL) (*p* < 0.001). No significant difference in Vitamin D levels was observed between males and females. Lower Vitamin D levels were significantly associated with advanced tumor grade (*p* = 0.004), lymphovascular invasion (*p* < 0.001), lymph node involvement (*p* = 0.001), and distant metastasis (*p* < 0.001). Representative histopathological images confirmed invasive moderately differentiated adenocarcinoma with characteristic malignant glandular architecture. Conclusions: Vitamin D deficiency was highly prevalent among colorectal cancer patients and was significantly associated with advanced tumor characteristics, including higher grade and metastatic features. These findings suggest a strong inverse relationship between serum Vitamin D levels and CRC development and progression. Further large-scale prospective and interventional studies are warranted to clarify the causal role of Vitamin D and its potential therapeutic implications in colorectal cancer prevention and management.

## 1. Introduction

Colorectal cancer (CRC) is one of the most common malignancies worldwide and ranks third in terms of both incidence and cancer-related mortality. It accounts for approximately 13% of all cancer diagnoses and represents the leading cancer of the gastrointestinal tract. Global projections estimate that by 2040, there will be approximately 3.2 million new CRC cases and 1.6 million related deaths, with the majority occurring in higher-income countries [1]. These alarming trends highlight the urgent need to identify modifiable risk factors associated with CRC development and progression.

In Saudi Arabia, CRC incidence varies across regions, with higher rates reported in Riyadh, Makkah, and the Eastern Province [2]. Epidemiological data indicate gender and age differences in disease presentation, with women often diagnosed at a younger age (45–59 years) compared to men (60–74 years) [3,4]. Despite these observations, the underlying biological and environmental factors contributing to regional and demographic variations remain incompletely understood.

Among the proposed modifiable risk factors, Vitamin D deficiency has gained significant attention. Numerous studies have demonstrated an association between low Vitamin D levels and an increased risk of various cancers, including CRC. In Saudi Arabia, Vitamin D deficiency is highly prevalent, affecting approximately 83.6% of the population [5], including university students who have demonstrated severe deficiency [6]. This widespread deficiency raises concerns regarding its potential role in the rising incidence of CRC in the region.

Vitamin D plays a critical role in maintaining intestinal homeostasis by regulating immune responses, reducing inflammation, preserving epithelial barrier integrity, and controlling cellular proliferation and differentiation. The biological effects of Vitamin D are mediated through the Vitamin D receptor (VDR), and deficiency in the Vitamin D/VDR pathway has been implicated in the pathogenesis of several diseases, including CRC [7,8]. Experimental evidence suggests that adequate Vitamin D levels modulate gene expression involved in inflammation, apoptosis, cell cycle regulation, and tumor suppression, thereby inhibiting cancer development and progression [9].

Growing epidemiological and clinical evidence supports an inverse relationship between circulating Vitamin D levels and CRC risk. Higher serum concentrations of 25-hydroxyvitamin D have been associated with a reduced risk of CRC development. A meta-analysis of 17 cohort studies reported that elevated circulating 25-hydroxyvitamin D_3_ levels were linked to a 19% reduction in CRC risk among women and a 7% reduction among men [10]. Furthermore, Vitamin D has been associated not only with reduced incidence but also with improved survival outcomes. Patients with stage III CRC in the highest quintile of Vitamin D levels demonstrated significantly better recurrence-free and overall survival compared with those in the lowest quintile [11].

Despite increasing evidence, data from the Saudi population—particularly regarding the relationship between Vitamin D status and clinicopathological characteristics of CRC—remain limited. Given the high prevalence of Vitamin D deficiency in Saudi Arabia and the rising burden of colorectal cancer, it is essential to investigate this association within the local context.

Therefore, the present study aimed to evaluate the relationship between serum Vitamin D levels and colorectal cancer, and to assess its association with demographic and clinicopathological features, including tumor grade, lymphovascular invasion, lymph node involvement, and metastasis. Understanding this relationship may contribute to improved risk stratification, preventive strategies, and potential therapeutic implications in CRC management.

## 2. Materials and Methods

This retrospective case–control study was conducted at the Department of Pathology of King Abdul-Aziz Specialist Hospital, Prince Muteb Hospital, and the Oncology Center in the Al Jouf region, Saudi Arabia. All colorectal specimens received between January 2021 and August 2024 were reviewed.

During the study period, 5399 gastrointestinal surgical and endoscopic specimens were processed. Of these, 2111 were colorectal specimens, and 107 were histopathologically diagnosed as colorectal cancer (CRC). Seven cases were excluded due to incomplete clinical or laboratory data, resulting in 100 newly diagnosed CRC cases eligible for analysis.

The control group consisted of 50 apparently healthy individuals recruited from hospital attendees undergoing routine health check-ups during the same study period and from the same geographic region. Controls were screened through medical record review to confirm absence of colorectal cancer, chronic systemic illness, malignancy at other sites, or Vitamin D supplementation.

Frequency matching was performed based on age group and gender to achieve comparable demographic distribution between cases and controls. Due to the retrospective design and limited availability of eligible controls, a 2:1 case-to-control ratio was used.

Inclusion criteria:

- Newly diagnosed colorectal cancer cases confirmed by histopathology;
- Patients of any age and gender;
- Patients diagnosed between January 2021 and August 2024.

Exclusion criteria:

- Patients who have received prior chemotherapy or radiotherapy;
- Malignancies originating outside the colon or rectum;
- Patients with chronic systemic illnesses;
- Patients receiving Vitamin D supplementation;
- Cases with incomplete clinical or laboratory data.

Ethical Approval:

Approved from the institutional review board IRB. For ethical consents, Hail Health Cluster Northern area, General Directorate for Research and Studies, Ministry of Health, was obtained to collect patients’ data on 9 September 2024, ethical code (IRB:2024-77). Moreover, informed consent was obtained from the healthy controls before participation in the study.

Data collection and analysis method:

Demographic data, histopathological findings (tumor subtype, differentiation grade, lymphovascular invasion, lymph node involvement, and metastasis), and serum Vitamin D levels were extracted from hospital records and recorded in a structured data sheet.

Data was analyzed using the Statistical Package for Social Sciences (SPSS), version 23. Descriptive statistics were presented as frequencies, percentages, means, and standard deviations.

Independent *t*-test was used to compare mean serum Vitamin D levels between CRC cases and controls. One-way ANOVA was used to assess differences in mean Vitamin D levels across age groups and tumor grades. Pearson chi-square test was used to assess associations between Vitamin D status categories and clinicopathological characteristics. Post hoc comparisons were performed using Tukey and LSD tests where appropriate.

To address potential confounding due to age and gender imbalance, multivariable logistic regression analysis was performed to evaluate the independent association between Vitamin D deficiency and colorectal cancer. Adjusted odds ratios (ORs) with 95% confidence intervals (CIs) were calculated. A *p*-value < 0.05 was considered statistically significant.

Given the retrospective nature of the study and fixed number of eligible cases, all available CRC cases during the study period were included. Although the case-to-control ratio was 2:1, statistical adjustment was performed to minimize potential bias arising from group imbalance.

Laboratory diagnosis:

Colorectal cancer diagnosis was confirmed through histopathological examination. Biopsy specimens were fixed in formalin, processed routinely, embedded in paraffin, sectioned, and stained with hematoxylin and eosin. Slides were independently reviewed and confirmed by two consultant pathologists.

For measurement of serum Vitamin D levels, blood samples were collected and centrifuged to separate serum. Serum 25-hydroxyvitamin D [25(OH)D] levels were measured using the ARCHITECT 25-OH Vitamin D reagent kit on the Architect i1000SR integrated immunoassay analyzer (Abbott, Dublin, Ireland).

Vitamin D status was classified as follows:

Deficiency: <20 ng/mL;

Insufficiency: 21–29 ng/mL;

Normal: ≥30 ng/mL.

Cut-off values were based on recommendations from the American College of Endocrinology and the Endocrine Society [12,13,14].

## 3. Results

During the 4-year study period, a total of 5399 gastrointestinal surgical and endoscopic specimens were processed. Of these, 2111 (39.1%) were colorectal specimens. Colorectal cancer (CRC) was diagnosed in 107 cases (5.1%). After excluding seven cases due to incomplete clinical or laboratory data, 100 newly diagnosed CRC patients were included in the final analysis. The control group consisted of 50 apparently healthy individuals.

The demographic distribution is presented in Table 1. The mean age of CRC patients was 53.07 ± 13.3 years (range: 20–71 years), while controls had a mean age of 49.8 ± 12.3 years (range: 20–68 years). The majority of CRC cases (69%) were aged > 50 years, indicating that CRC predominantly affected older individuals. Among cases, males represented 58% and females 42%, resulting in a male-to-female ratio of 1.4:1. Most patients were Saudi nationals (82%). These findings confirm that CRC in this cohort was more frequent in older males, consistent with established epidemiological trends.

The comparison of mean serum Vitamin D levels across age groups is shown in Table 2. The highest mean Vitamin D level was observed in the 20–30-year group (31.10 ± 18.72 ng/mL), whereas the lowest mean level was found in individuals aged > 50 years (20.99 ± 12.76 ng/mL). One-way ANOVA revealed a statistically significant difference among age groups (F(3,146) = 3.295, *p* = 0.022), indicating that Vitamin D levels decreased significantly with increasing age.

Vitamin D distribution between males and females is summarized in Table 3. Deficiency (<20 ng/mL) was observed in 46.6% of males and 46.8% of females, showing similar prevalence between genders. The mean Vitamin D levels were comparable between males (24.17 ± 15.80 ng/mL) and females (23.98 ± 13.84 ng/mL). Independent *t*-test analysis demonstrated no statistically significant difference between sexes (t = 0.074, df = 148, *p* = 0.941). These results indicate that gender was not significantly associated with Vitamin D levels in this cohort.

**Figure 1:**
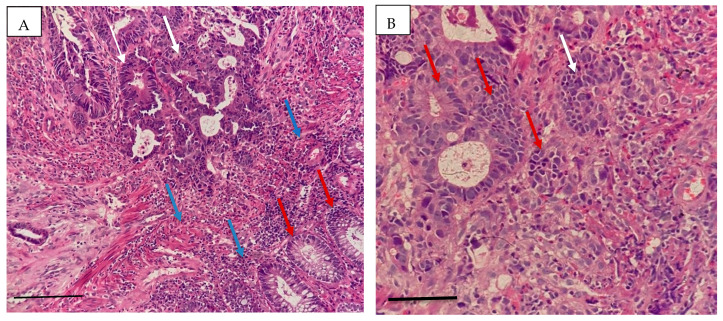
((**A**) scale bar= 200 μm 10×, (**B**) scale bar= 50 μm 40×) Invasive moderately differentiated adenocarcinoma of the rectum. The tumor is composed of irregular glands with complex branching, budding, and a cribriform pattern, all lined by dysplastic epithelium (white arrow). Epithelial cells exhibit loss of mucin, nuclear hyperchromasia, stratification, and increased mitosis (red arrow). The stroma is infiltrated by inflammatory cells, accompanied by a desmoplastic reaction (blue arrow).

**Figure 2:**
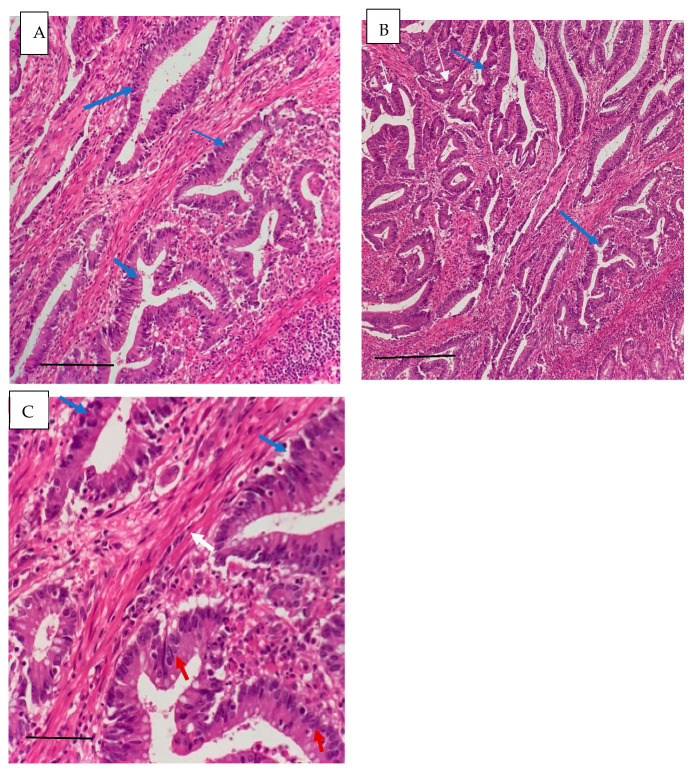
((**A**) scale bar =200 μm 20×, (**B**) scale bar= 200 μm 10×, (**C**) scale bar= 50 μm 40×): Moderately differentiated adenocarcinoma of the right ascending colon. The sections show an infiltrative tumor composed of complex glands (blue arrow). The tumor cells are pleomorphic, with eosinophilic cytoplasm, hyperchromatic oval nuclei, and prominent nucleoli. (red arrow). The tumor infiltrates the mucosa, submucosa, and muscularis propria (white arrow).

The histopathological characteristics are presented in Table 4. Adenocarcinoma was the predominant subtype (81%), most tumors were moderately differentiated (56%), the sigmoid colon (39%) was the most common tumor location, and Lymphovascular invasion (37%), lymph node involvement (31%), and metastasis (19%) were observed. These findings indicate that a considerable proportion of patients presented with advanced pathological features. The histopathological characteristics of colorectal cancer were confirmed by microscopic examination. Representative images are shown in Figure 1 and Figure 2.

As shown in Table 5, CRC patients had significantly lower serum Vitamin D levels compared to controls. Mean Vitamin D in cases: 18.7 ± 11.3 ng/mL; Mean Vitamin D in controls: 34.9 ± 15.6 ng/mL; and Independent *t*-test: t = −6.58, df = 75.64, *p* < 0.001. Vitamin D deficiency was present in 59% of CRC cases compared to only 22% of controls, demonstrating a strong inverse association between Vitamin D status and colorectal cancer.

The relationship between serum Vitamin D levels and tumor aggressiveness is presented in Table 6. A significant inverse association was observed between Vitamin D levels and tumor grade, with progressively lower mean levels in well-differentiated tumors (22.08 ± 8.50 ng/mL), moderately differentiated tumors (18.67 ± 12.65 ng/mL), and poorly differentiated tumors (9.50 ± 4.75 ng/mL). This difference was statistically significant (F(2,97) = 5.925, *p* = 0.004). Similarly, patients with positive lymphovascular invasion (LVI) had significantly lower Vitamin D levels (13.62 ± 7.55 ng/mL) compared to those without LVI (21.63 ± 12.13 ng/mL) (*p* < 0.001). Mean Vitamin D levels were also significantly reduced in patients with lymph node involvement (12.95 ± 7.99 ng/mL) compared to those without involvement (21.24 ± 11.68 ng/mL) (*p* = 0.001). Furthermore, patients with distant metastasis exhibited markedly lower Vitamin D levels (10.51 ± 5.04 ng/mL) than non-metastatic patients (20.58 ± 11.54 ng/mL) (*p* < 0.001). Overall, these findings demonstrate a consistent and statistically significant inverse relationship between serum Vitamin D levels and increasing tumor aggressiveness.

## 4. Discussion

Colorectal cancer (CRC) remains one of the leading causes of cancer-related morbidity and mortality worldwide and across the Arab region. In Saudi Arabia, CRC is the most common malignancy among men and the third most common among women, with a noticeable increase in incidence among older individuals. Increasing epidemiological evidence has highlighted the association between Vitamin D deficiency and several malignancies, including CRC.

In the present study, the mean age of CRC patients was 53.07 ± 13.3 years, which is consistent with previous findings reporting similar age distributions [15]. The majority of cases (69%) occurred in individuals aged over 50 years, supporting earlier reports indicating that most CRC cases are diagnosed in older populations [16]. These findings reinforce the well-established relationship between advancing age and CRC risk.

A male predominance was observed (58%), with a male-to-female ratio of 1.4:1, which aligns with regional studies reporting similar gender distributions [15,16]. Additionally, the majority of patients were Saudi nationals (82%), consistent with previous local data [17].

Histopathologically, invasive adenocarcinoma was the most frequent subtype (81%), followed by mucinous adenocarcinoma (7%) and squamous carcinoma (12%). These results are comparable to those of earlier studies reporting adenocarcinoma as the dominant histological type in CRC [16,18]. The sigmoid colon was the most common tumor location (39%), which is consistent with findings from regional studies [17], although some studies have reported rectal predominance [19].

Regarding tumor differentiation, moderately differentiated tumors were the most common (56%), followed by well-differentiated (32%) and poorly differentiated tumors (12%) [20]. Lymphovascular invasion was present in 37% of cases, lymph node involvement in 31%, and distant metastasis in 19%. These findings are comparable to those in previous reports from Saudi Arabia, although slightly lower metastatic rates have been documented in some studies [19,21].

A major finding of this study is the significant inverse association between serum Vitamin D levels and CRC. The mean Vitamin D level in CRC patients (18.7 ± 11.3 ng/mL) was significantly lower than in controls (34.9 ± 15.6 ng/mL) (*p* < 0.001). Furthermore, Vitamin D deficiency was significantly more prevalent among cases (59%) compared to controls (22%). These findings strongly support previous studies demonstrating an inverse relationship between serum Vitamin D levels and CRC risk [22,23]. The marked difference between cases and controls reinforces the potential protective role of Vitamin D in colorectal carcinogenesis.

Although no statistically significant association was observed between Vitamin D levels and gender, age-related analysis demonstrated significantly lower Vitamin D levels in older age groups (>50 years), consistent with previous reports [24]. The reduction in Vitamin D levels with increasing age may be attributed to reduced sun exposure and lifestyle-related factors. While males showed slightly higher deficiency rates than females, the difference was not statistically significant, consistent with variable findings in previous studies [8,25].

Importantly, Vitamin D deficiency was significantly associated with tumor aggressiveness. Mean Vitamin D levels decreased progressively with increasing tumor grade, and the association was statistically significant (*p* = 0.004). Poorly differentiated tumors exhibited the lowest Vitamin D levels. This observation is consistent with evidence suggesting that well-differentiated colorectal cancers demonstrate stronger Vitamin D receptor (VDR) expression compared to poorly differentiated tumors [26]. Reduced VDR expression in advanced tumors may impair the anti-proliferative and differentiation-promoting effects of Vitamin D.

Additionally, significant inverse associations were found between Vitamin D levels and lymphovascular invasion, lymph node involvement, and distant metastasis (all *p* ≤ 0.001). Notably, metastatic cases showed markedly reduced Vitamin D levels. These findings are consistent with experimental evidence indicating that Vitamin D may inhibit tumor proliferation, invasion, angiogenesis, and metastatic progression [27]. The strong and consistent associations across multiple clinicopathological parameters suggest that Vitamin D deficiency may be linked not only to CRC occurrence but also to tumor progression and severity.

Although this study has limitations, including its retrospective design and sample size, the consistent findings across demographic, biochemical, and pathological parameters strengthen the validity of the results. However, causality cannot be inferred from this study. Future prospective cohort studies and randomized controlled trials are necessary to clarify whether Vitamin D supplementation can reduce CRC risk or improve clinical outcomes.

In conclusion, this study demonstrates a significant inverse association between serum Vitamin D levels and colorectal cancer, particularly with advanced tumor grade and aggressive pathological features. These findings highlight the potential clinical importance of Vitamin D assessment in CRC patients and support further mechanistic and interventional research in this field.

## 5. Conclusions and Recommendations

This study demonstrates a significant inverse association between serum Vitamin D levels and colorectal cancer (CRC). CRC was more frequently diagnosed in males and predominantly affected individuals over the age of 50 years. A substantial proportion of patients exhibited Vitamin D deficiency, which was significantly associated with advanced tumor grade, lymph node involvement, lymphovascular invasion, and distant metastasis. Notably, lower Vitamin D levels were consistently observed in patients with more aggressive clinicopathological features.

These findings suggest that Vitamin D deficiency may be associated not only with CRC risk but also with tumor progression and disease severity. The strong relationship between deficient Vitamin D status and adverse pathological characteristics supports the hypothesis that Vitamin D may play a modulatory role in colorectal carcinogenesis and tumor differentiation. Although causality cannot be established due to the retrospective design, the observed associations highlight the potential clinical relevance of Vitamin D status in CRC patients.

Based on the findings of this study, routine screening for Vitamin D deficiency in individuals at risk for colorectal cancer, particularly those over 50 years of age, should be considered. Public health initiatives promoting adequate sun exposure, dietary intake of Vitamin D–rich foods, and appropriate supplementation for deficient individuals are recommended. Additionally, well-designed prospective and interventional studies are needed to determine whether optimizing Vitamin D levels can contribute to colorectal cancer prevention and improve clinical outcomes.

Limitations: While this study provides meaningful insights into the association between Vitamin D deficiency and colorectal cancer, certain limitations should be acknowledged. The relatively modest sample size and retrospective design may limit the ability to establish a definitive causal relationship. Additionally, some potential confounding factors such as lifestyle patterns, sunlight exposure, and body mass index were not comprehensively assessed. Nevertheless, the consistent and statistically significant associations observed across multiple clinicopathological parameters strengthen the reliability of the findings and provide a solid foundation for future large-scale prospective investigations.

## Figures and Tables

**Table 1:** Age and gender distribution of cases and controls.

Age Group	Cases (n = 100)		Control (n = 50)	
	Male (n = 58)	Female (n = 42)	Male (n = 30)	Female (n = 20)
30 years (n = 14)	3 (75)	1 (25)	7 (70)	3 (30)
31–40 years (n = 21)	6 (50)	6 (50)	4 (44.4)	5 (55.6)
41–50 years (n = 29)	10 (66.7)	5 (33.3)	9 (64.3)	5 (35.7)
>50 years (n = 86)	39 (56.5) *	30 (43.5)	10 (58.8) *	7 (41.2)

* Denotes highest frequency within group.

**Table 2:** Mean serum Vitamin D levels by age group (n = 150).

Age Group (Years)	n	Mean Vitamin D (ng/mL)	SD
20–30	14	31.10	18.72
31–40	21	26.44	14.57
41–50	29	28.21	17.58
>50	86	20.99	12.76
Total	150	—	—

ANOVA result: F(3,146) = 3.295, *p* = 0.022.

**Table 3:** Comparison of Vitamin D status between males and females.

Study Population	Normal (≥30 ng/mL)	Insufficient (21–29 ng/mL)	Deficient (<20 ng/mL)	Mean Vitamin D (ng/mL) ± SD	Independent *t*-Test
**Male (n = 88)**	27 (30.7%)	20 (22.7%)	41 (46.6%)	24.17 ± 15.80	t = 0.074
**Female (n = 62)**	16 (25.8%)	17 (27.4%)	29 (46.8%)	23.98 ± 13.84	df = 148
					*p* = 0.941

**Table 4:** Clinicopathological features of CRC patients (n = 100).

Characteristic	Male (n = 58)	Female (n = 42)	Total (n = 100)
**Histological Type**			
Adenocarcinoma	50 (86.2%)	31 (73.8%)	81 (81%)
Mucinous adenocarcinoma	3 (5.2%)	4 (9.5%)	7 (7%)
Squamous carcinoma	5 (8.6%)	7 (16.7%)	12 (12%)
**Tumor Grade**			
Well differentiated	17 (29.3%)	15 (35.7%)	32 (32%)
Moderately differentiated	32 (55.2%)	24 (57.1%)	56 (56%)
Poorly differentiated	9 (15.5%)	3 (7.2%)	12 (12%)
**Tumor Location**			
Sigmoid colon	25 (43.1%)	14 (33.3%)	39 (39%)
Ascending colon	7 (12.1%)	4 (9.5%)	11 (11%)
Caecum	4 (6.9%)	2 (4.8%)	6 (6%)
Rectum	6 (10.3%)	9 (21.4%)	15 (15%)
Colon & rectum	10 (17.2%)	9 (21.4%)	19 (19%)
Transverse colon	1 (1.7%)	1 (2.4%)	2 (2%)
Descending colon	5 (8.6%)	3 (7.1%)	8 (8%)
**Lymphovascular Invasion (LVI)**			
Positive	20 (34.5%)	17 (40.5%)	37 (37%)
Negative	38 (65.5%)	25 (59.5%)	63 (63%)
**Lymph Node Involvement (LNI)**			
Positive	23 (39.7%)	8 (19.0%)	31 (31%)
Negative	35 (60.3%)	34 (81.0%)	69 (69%)
**Metastasis**			
Positive	12 (20.7%)	7 (16.7%)	19 (19%)
Negative	46 (79.3%)	35 (83.3%)	81 (81%)

**Table 5:** Comparison of Vitamin D status between CRC cases and controls.

Study Population	Normal (≥30 ng/mL)	Insufficient (21–29 ng/mL)	Deficient (<20 ng/mL)	Mean Vitamin D (ng/mL) ± SD	Independent *t*-Test
**Cases (n = 100)**	10 (10%)	31 (31%)	59 (59%)	18.7 ± 11.3	t = −6.58
**Controls (n = 50)**	33 (66%)	6 (12%)	11 (22%)	34.9 ± 15.6	df = 75.64
					*p* < 0.001

**Table 6:** Association between Vitamin D levels and tumor characteristics (n = 100).

Cancer Characteristic	Group (n)	Mean Vitamin D (ng/mL)	SD	ANOVA
**Tumor Grade**				
Well differentiated (32)	32	22.08	8.50	F(2,97) = 5.925
Moderately differentiated (56)	56	18.67	12.65	*p* = 0.004
Poorly differentiated (12)	12	9.50	4.75	
**Lymphovascular Invasion (LVI)**				
Present (37)	37	13.62	7.55	F(1,98) = 13.124
Absent (63)	63	21.63	12.13	*p* < 0.001
**Lymph Node Involvement (LNI)**				
Present (31)	31	12.95	7.99	F(1,98) = 12.853
Absent (69)	69	21.24	11.68	*p* = 0.001
**Metastasis**				
Present (19)	19	10.51	5.04	F(1,98) = 13.764
Absent (81)	81	20.58	11.54	*p* < 0.001

## Data Availability

No new data was created.
